# Supplementary material for: Enhanced superconductivity accompanying a Lifshitz transition in electron-doped FeSe monolayer
Source: Nat Commun. 2017 Apr 19;8:14988. doi: 10.1038/ncomms14988 (PMC5399296; doi:10.1038/ncomms14988)
Supplement: Supplementary Information — Supplementary Figures and Supplementary References [file ncomms14988-s1.pdf]

Supplementary figures

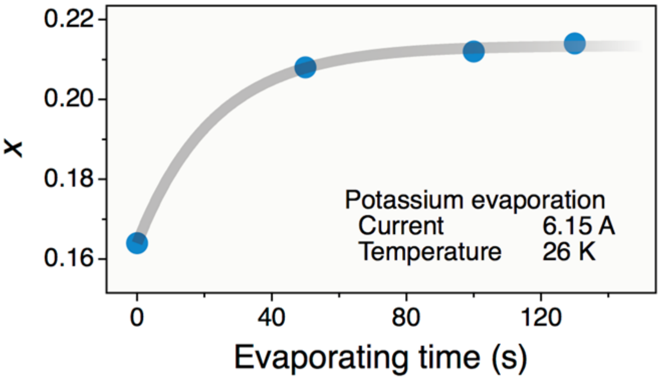

Supplementary Figure 1 | Evolution of the electron doping level upon K evaporation. The grey line is a guide for the eye to show how the system evolves towards saturation upon consecutive K evaporation. Previous STM studies reported that the K atoms stay on the surface of the film [1, 2, 3]. Parts (could be all with very low coverage) of them are ionized, as supported by the observation of the K atom clusters with increasing coverage (>0.2ML) in the STM images. Unfortunately, we are not equipped with a low-temperature STM with atomic resolution. However, our ARPES (a sophisticated version of photoemission compared to XPS) data clearly show a chemical potential shift, thus indicating electron-doping in our experiment. The extra electrons can only come from the ionization of K.

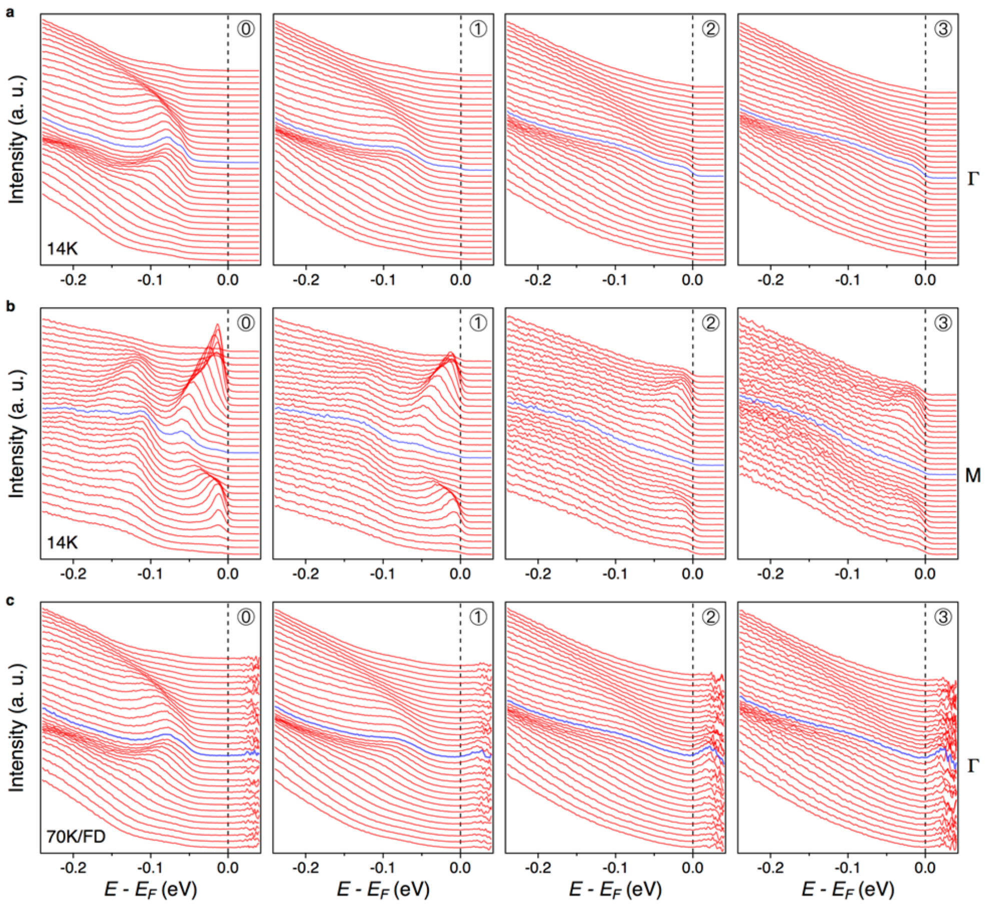

Supplementary Figure 2 | Evolution of the energy distribution curves (EDCs) upon K deposition. a-b, Potassium coating evolution of the EDC plots at 14 K near Γ and M along the direction shown in the inset of Fig. 1 **e**. **c,** EDC plots along the same cut as in **a**, but recorded at 70 K. The EDCs are divided by the Fermi-Dirac distribution function convoluted by the resolution function to visualize the states above E_{F}.

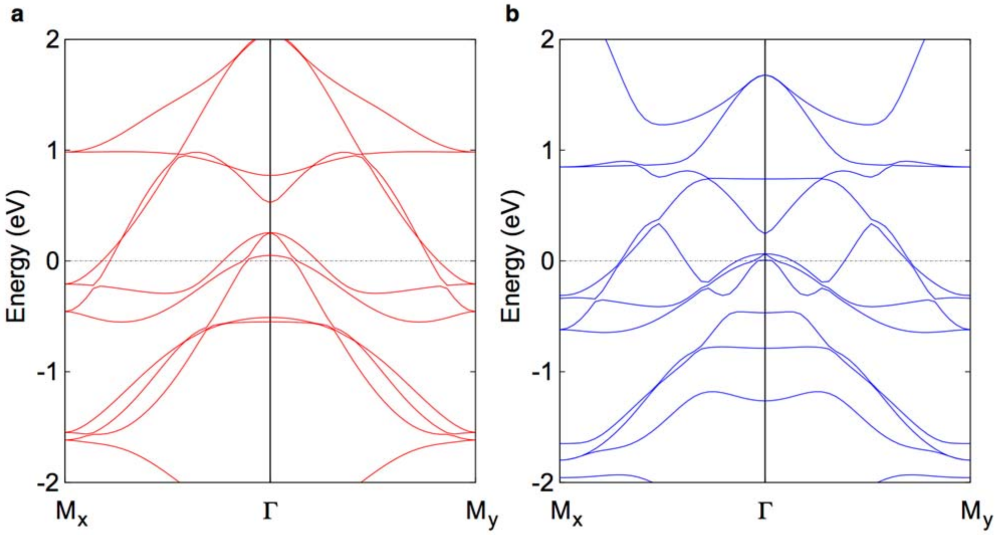

c

Atomic positions	FeSe	K-FeSe
Fe	(0,0,0) / (1.9,1.9,0)	(0,0,0) / (1.9,1.9,0)
Se (top)	(1.9,0,1.41)	(1.9,0,1.4144)
Se (bottom)	(0,1.9,-1.41)	(0,1.9,-1.4041)
K	/	(0,1.9,3.333)

Supplementary Figure 3 | LDA band structures. a, Calculated electronic band structure of pristine monolayer FeSe. **b,** Calculated electronic band structure of FeSe topped by one layer of K, with one K atom per unit cell of FeSe. The gap between the d_{xy}/p_z band and the d_{xz}/d_{yz} band at Γ decreases from 270 to 180 meV with K deposition, and there is no band related to K nearby. In both cases, the lattice constant is 3.8 Å and the internal atomic positions are fully relaxed. **c,** Atomic positions of the pristine and K topped monolayer FeSe. In the former, the bond length of Fe-Se is 2.366. In the latter, the bond lengths of Fe-Se(top) and Fe-Se(bottom) are 2.36866 and 2.36250, respectively.

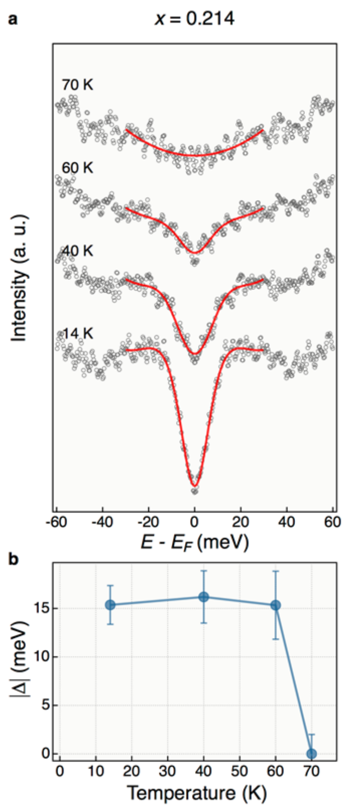

Supplementary Figure 4 | Superconducting gap of potassium-coated 1UC FeSe/STO with the doping level of x = 0.214. a, Temperature evolution of the symmetrized EDCs at the k_F point of the electron FS around M. The red curves correspond to fit of the data. **b,** Superconducting gap sizes as a function of temperature obtained from the fits shown in **a**. Error bars are estimated from the standard deviation of the fitting.

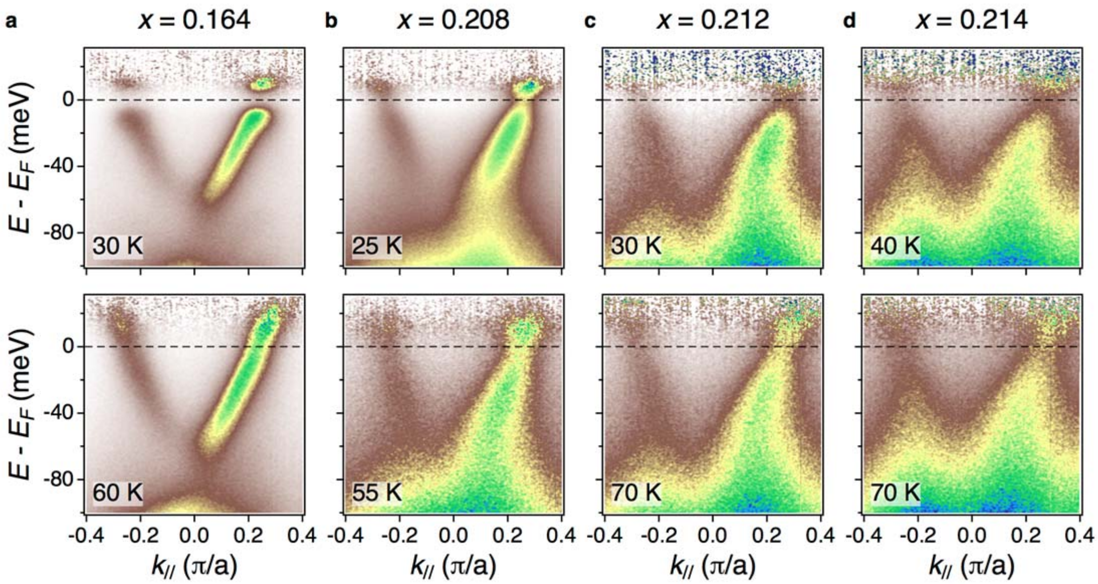

Supplementary Figure 5 | Comparison of the spectra at different temperatures for each sample. a, ARPES intensity plots near the M point recorded at the low and high temperature for the pristine 1UC FeSe/STO. The spectra are divided by the Fermi-Dirac function in order to access partly the unoccupied states. **b-d,** Same as **a** but for the potassium-coated film with the electron-doping level indicated above the panels, respectively.

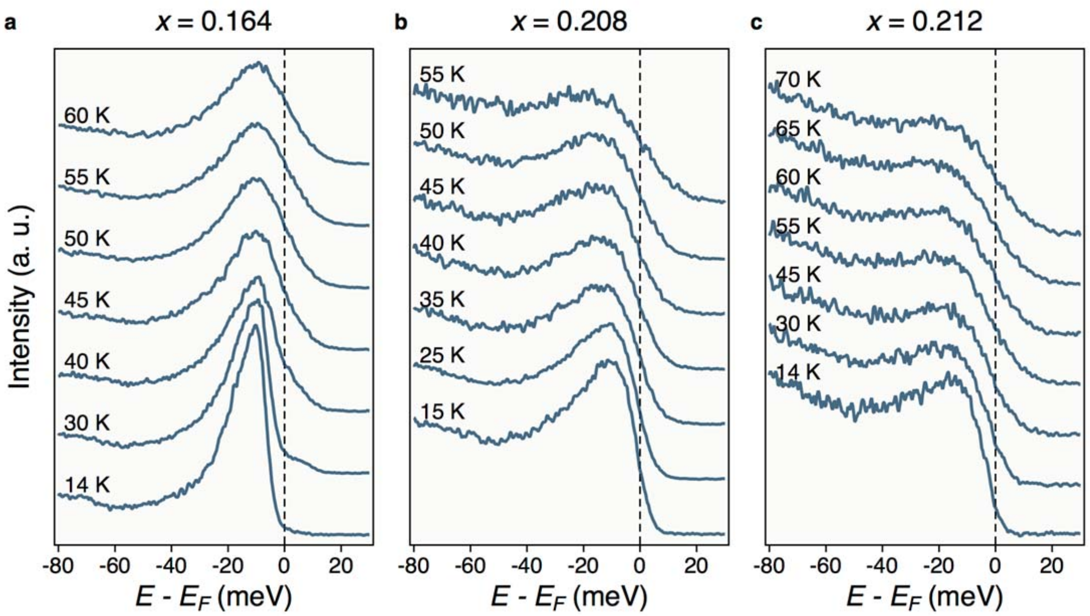

Supplementary Figure 6 | Temperature evolution of the EDCs. a-c, Temperature evolution of the EDCs at the k_F point of the electron FS around M for pristine and potassium-coated 1UC FeSe/STO. The electron doping is indicated above the panels.

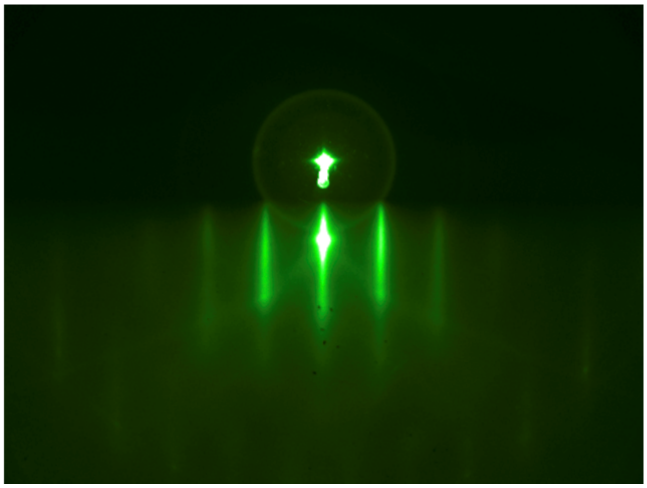

Supplementary Figure 7 | Refection high-energy electron diffraction (RHEED) image. RHEED image of the pristine monolayer film of FeSe/STO after annealing at 350 ℃ for 20h.

Supplementary References

1. Song, C.-L. *et al*. Observation of Double-Dome Superconductivity in Potassium-Doped FeSe Thin Films. *Phys. Rev. Lett.* **116**, 157001 (2016).

2. Tang, C. *et al*. Interface-enhanced electron-phonon coupling and high-temperature superconductivity in potassium-coated ultrathin FeSe films on $SrTiO_3$. *Phys. Rev. B* **93**, 020507(R) (2016).

3. Zhang, W. H. *et al*. Effects of Surface Electron Doping and Substrate on the Superconductivity of Epitaxial FeSe Films. *Nano Lett.* **16**, 1969 (2016).
